# The impact of surge adaptations on hospitalist care teams during the COVID-19 pandemic utilizing a rapid qualitative analysis approach

**DOI:** 10.1186/s13690-022-00804-7

**Published:** 2022-02-17

**Authors:** Angela Keniston, Vishruti Patel, Lauren McBeth, Kasey Bowden, Alexandra Gallant, Marisha Burden

**Affiliations:** 1 Division of Hospital Medicine, University of Colorado School of Medicine, Aurora, USA; 2 University of Colorado School of Medicine, Aurora, USA

**Keywords:** COVID, Surge, Hospitalist, Qualitative research, Rapid qualitative methods

## Abstract

**Background:**

Hospital systems have rapidly adapted to manage the influx of patients with COVID-19 and hospitalists, specialists in inpatient care, have been at the forefront of this response, rapidly adapting to serve the ever-changing needs of the community and hospital system. Institutional leaders, including clinical care team members and administrators, deployed many different strategies (i.e. adaptations) to manage the influx of patients. While many different strategies were utilized in hospitals across the United States, it is unclear how frontline care teams experienced these strategies and multifaceted changes. As these surge adaptations likely directly impact clinical care teams, we aimed to understand the perceptions and impact of these clinical care and staffing adaptations on hospitalists and care team members in order to optimize future surge plans.

**Methods:**

Qualitative, semi-structured interviews and focus groups with hospitalist physicians, advanced practice providers (APPs), and hospital nursing and care management staff at a quaternary academic medical center. Interviews focused on the impact of COVID-19 surge practices on the following areas: (1) the experience of clinical care teams with the adaptations used to manage the surge (2) the perception and experience with the communication strategies utilized (3) the personal experience with the adaptations (i.e. how they impacted the individual) and (4) if participants had recommendations on strategies for future surges. We utilized rapid qualitative analysis methods to explore themes and subthemes.

**Results:**

We conducted five focus groups and 21 interviews. Three themes emerged from the work including (1) dynamic clinical experience with a lot of uncertainty, (2) the importance of visible leadership with a focus on sense-making, and (3) the significant emotional toll on care team members. Subthemes included sufficient workforce, role delineation and training, information sharing, the unique dichotomy between the need for flexibility and the need for structure, the importance of communication, and the emotional toll not only on the provider but their families. Several recommendations came from this work.

**Conclusions:**

COVID-19 surge practices have had direct impact on hospitalists and care team members. Several tactics were identified to help mitigate the many negative effects of COVID-19 on frontline hospitalist providers and care teams.

**Supplementary Information:**

The online version contains supplementary material available at 10.1186/s13690-022-00804-7.

## Background

The COVID-19 pandemic has led to waves of large influxes of patients requiring hospitalization. Hospitalists, which are specialists in inpatient medicine, have been at the forefront of the COVID-19 pandemic, providing inpatient care for patients with COVID-19 and also leading hospitalist, hospital, and system-level adaptations in response to the growing pandemic [1, 2] in partnership with nursing, social work, and other frontline care team members. As the threat of COVID-19 has loomed over our healthcare systems, rapid and large-scale deployment of providers, novel ways to provide care, and information sharing occurred [1, 3, 4].

While previous disaster planning efforts have focused on large-scale (but short term) disasters [5], the COVID-19 pandemic represents a longer-term challenge with intermittent but large fluctuations in patients with COVID-19 (i.e. surges, periods of time with large numbers of patients being diagnosed with COVID-19 resulting in increased hospitalizations) [1]. Because of the rapidly evolving situation, many changes had to occur in how frontline care team members provided patient care (i.e. adaptations). Role shifting, training of new care team members, and team adaptations had to occur quickly with large-scale communication cascades. Examples of the adaptations included hospitalist providers (physicians and advanced practice providers such as physician assistants and nurse practitioners) increasingly caring for critically ill patients, outpatient providers moving from the outpatient setting to the inpatient clinical setting to help manage the large influx of patients, and streamlining of communication strategies in response to the sheer quantity and dynamism of the situation. Additionally, care team members such as social workers and case management (members of the team who help to coordinate care during and after hospitalization) had to rapidly adapt to different regulatory requirements and shifting situations regarding to where patients could discharge. One early example during the pandemic was that patients who needed ongoing rehabilitation care did not have facilities that they could go to because of the highly infectious nature of COVID-19 and the lack of information at the time around safety of transferring patients to these types of facilities. These are just a few examples of how frontline care teams were impacted.

In addition to clinical care, hospitalists are also hospital systems experts and have a number of tools to evaluate processes, experiences, and outcomes in the complex and dynamic clinical environment of the hospital, namely traditional quantitative research methodologies, quality and process improvement, and qualitative and mixed methods approaches. However, some of these traditional methods and the findings that result from them may lag in comparison to the fluid and dynamic environment in which hospitalists work. The COVID-19 pandemic highlighted the need for rapid yet methodologically sound methods to understand and drive complex systems changes.

To date, there have been some descriptions of adaptations utilized by hospitalists and hospitals [1, 2, 4, 6], however, the experience with and perceived impact of these adaptations is unknown. Recognizing that the frontline workforce experienced these adaptations on a daily basis, we conducted qualitative, semi-structured interviews and focus groups with hospitalist physicians, advanced practice providers (APPs), and hospital nursing and care management staff following the first COVID surge in order to better understand the experience of and perceived impact of the adaptations and to prepare for future surges.

## Methods

### Study design

We conducted a program evaluation with semi-structured interviews and focus groups with hospitalist physicians and APPs, care management (including care managers and social workers), and nursing in a single quaternary academic medical center from September 11, 2020 to October 23, 2020. Semi-structured interviews were chosen to help provide guidance on the topic content but to also allow for the generation of new ideas and thought around the various adaptations implemented. We recruited providers and care team members including hospitalist physicians, APPs, social workers, case managers, and nurses as many of the adaptations deployed by our hospitalist team had wide reaching impact. We needed to understand the impact on the hospitalist team as well as others; thus a multidisciplinary approach was taken. This project was approved by the Colorado Multiple Institutional Review Board and was deemed non-human subject research (COMIRB 20–2242).

### Setting and participants

Hospitalist providers were recruited through email advertisements as well as presentations at meetings. These invitations were sent to the entire team (~ 100 faculty that included physicians and APPs). Staff in care management and nursing leadership also received email invitations. Targeted emails were sent to individuals that were felt to have significant insights and experiential knowledge of the COVID surge plans. Individuals did not receive more than three emails per our research board guidelines. In order to ensure confidentiality, no additional information was collected from the participants.

Our original surge plan has been previously described in detail [1]. See Appendix Fig. 1 for high-level framework for surge plans. To give context to the type of surge experienced during this study period, the highest census of patients with COVID was 145 (hospital size is approximately 678 beds).

**Fig.1:**
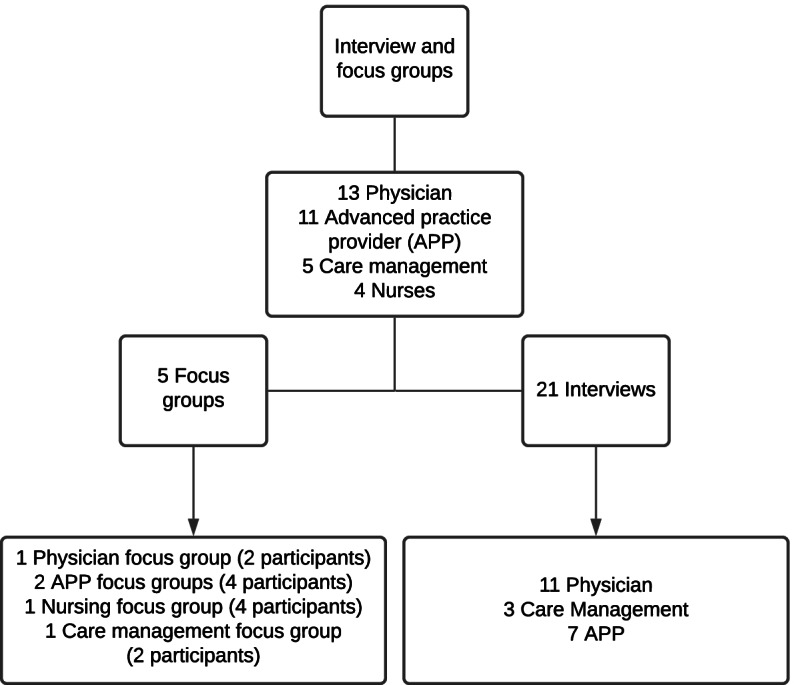
Enrollment

### Interview guide

Semi-structured interviews addressed the impact of COVID-19 surge practices in the following domains: (1) clinical experience, (2) communications during the surge, (3) the personal experience of COVID-19, and (4) recommendations for the future. Questions were derived through a literature review as well as based upon the expertise and objectives of the hospital medicine clinical operations leadership team. The questions were reviewed and iteratively developed by the study leadership team. Once focus groups were started the questions did not need further revisions. Because the study approach was semi-structured, the interviewers were able to bring up additional questions based upon what the participants said. Questions were modified for the care management, social work, and nurse leaders and were aimed at understanding team collaboration with the hospitalist group. The full interview guides are available in the Appendix 2.

Focus groups were conducted with individuals from the same profession/training background (i.e. there were specific focus groups for physicians, for advanced practice providers, and for care management which consisted of social work and case managers). Groups were grouped as such in order to limit any power dynamics that might inadvertently affect participants’ willingness to share their experiences.

### Data collection

Eligible participants were consented and interviewed by investigators (A.K., V.P., L.M., and A.G.). The investigators who conducted interviews were not involved in the development of surge plans thus limiting the potential to influence the direction of the conversation. Recruitment of participants was halted when no new codes or themes emerged during analysis. Interviews and focus groups were audio-recorded, de-identified, and transcribed. Any identifiers inadvertently captured on the audio-files were removed during professional transcription. The interview transcripts were supplemented with notes and observations by research personnel based off tone and other non-transcribed factors (A.K., V.P., L.M., and A.G.). The transcripts were also reviewed for completeness.

### Analysis

After professional transcription, a rapid qualitative analysis process [7, 8] was utilized. Rapid qualitative methods have been previously described, consisting of a structured analysis that can be useful in situations of dynamic, rapidly changing real-world situations (such as a pandemic) [9]. Templated summaries were created for each of the interviews and focus groups using Microsoft Word. One summary was created for each focus group or interview. Each summary consisted of a bullet-pointed list rather than complete sentences organized based on domains (or key topics). The bullet points did not represent any interpretation but instead, summarized the content of the interview/focus group. Each team member summarized the same transcript (A.K., V.P, L.M., K.B., A.G., M.B.) and then compared across team members in order to standardize the summary template methodology. Once this process was complete, five team members (V.P, L.M, K.B., A.G, and M.B) completed summaries for each interview/focus group. The remaining team member (A.K.) then reviewed each summary and associated transcript to confirm concordance in methods applied.

A matrix analysis was then conducted. A matrix is a tool that allows for an organized display of summarized data. Matrix analysis is an analytic tool that utilizes the organization of a matrix and the flow within a table to begin to understand what themes may exist within the data and how the data interact [8]. Considering the various domains or subgroups of interest, we decided which domains to analyze first. We constructed a matrix by copying and pasting the content of the summaries for each domain into an Excel file. Each row in a matrix is content from a given interview/focus group and each column refers to a unique domain or question from the interview guide. Once all summary content was copied over, we looked across rows, down columns, and then in a zig-zag pattern to begin to identify recurring concepts and patterns [10]. The matrices were analyzed by each team member and insights were discussed until consensus was reached and themes and sub-themes were emerged (A.K., V.P, L.M., K.B., A.G., M.B.). We utilized an inductive approach [11] allowing the concepts, patterns, and ultimately themes to emerge from the data rather than a deductive approach given there has not been (to date) much published on this topic area. Member checking [12], which is a technique for exploring the credibility of results, was conducted and no significant revisions were suggested.

## Results

We conducted five focus groups and 21 interviews (a total of 33 individuals, including 13 physicians, 11 APPs, 4 nurses, 2 social workers, and 3 care managers) **(**Fig. 1**)**. Three themes emerged from the work including (1) dynamic clinical experience with a lot of uncertainty (2) the importance of visible leadership with a focus on sense-making and (3) the significant emotional toll on care team members. Additional subthemes are highlighted below. Table 1–3 highlights exemplar quotes from each of the themes/subthemes.

**Table 1:** Dynamic clinical experience subthemes and exemplar quotes

Subtheme	Quotes
**Staffing concerns and ensuring sufficient workforce**	“And so the days were a little bit long and exhausting in the fact that it really took some mind—and some energy essentially, so mental energy to do that. Not only are you thinking about all the clinical stuff, but then you're also thinking about all of your actions and your movements, which you're not normally doing” (APP, Interview/FG 19) “I could imagine this having gone one of two ways. One where we asked providers, hospitalists to do more, meaning if I work, 14 days a month then I'm going to work 21, I’m going to work an extra week because we don’t have enough staff. There was never an assumption that there was—Almost like never an expectation that we would have to work extra shifts, what the division did, was they set up help from outside the division. And I think, when it comes to well-being, one, not overworking the provider and making sure the provider is being heard, are two most important aspects. I'm not a big believer that doing yoga in the office is going to improve your wellbeing if you’re working a ton.” (MD/DO, Interview/FG 25) "Hey, like we need to be prepared for this, this, and this. We're going to potentially surge you to the intensive care unit. We're going to potentially surge you to X, Y, or Z" It was like all these potentials, which was great to talk about, but at the same time before it happened, you're gasping, "Oh my god, like how bad is this going to get?". And then, you know, you're just hearing all this stuff. So, you know, the initial anxiety level was out of this world. (APP, Interview/FG 4) “We were willing to be moldable, and I think like that was really key, like everyone just being like we’re all in this together and even though we only have the schedule one or two weeks out, we don’t really know what things are going to look like. We’re all like on the same team, and I think sometimes there was maybe too much of trying to predict what all things are going look like and not just be like let’s just see how things evolve, but that – I think that’s a balance. Right? Because people want to have some predictability to their schedule.” (APP, Interview/FG 6)
**Role delineation and appropriate training/working to top of license**	“Splitting things from a provider side of things is really great, because it took a lot of the burden off and I very much trust my APP colleagues to, you know, go in and examine a patient. Again, they were, they're the experts in dealing with COVID patients.” (MD/DO, Interview/FG 13) “COVID surge was when nursing got flipped from—they pulled ortho nursing into, taking care of these COVID patients. I mean that was like a whole new learning curve for them. Getting comfortable, again, with the communication expectations from nursing and us was just a little bit of learning curve because this wasn't a group that we normally work with. I had lots of conversations with, floor managers, nursing managers about expectations because we were getting calls constantly about these patients. And some of them, you know, really legitimate and some of them were like, you know, as the days and weeks like, no, this is just a COVID patient, sadly.” (APP, Interview/FG 4)
**Information sharing – centralized hubs and colleagues as resources**	“The up-to-date summary. I reviewed that every time before I went on service. I reviewed the latest one, in addition to other literature, but it was stellar. And the ability to have someone to synthesize stuff as things went on, I knew that I had a document I could go to about best practices was really helpful.” (MD/DO, Interview/FG 1) “And so it was important to have those daily updates or, frequent updates, and then, the call-in meetings that we'd have sometimes multiple times a day, with clinical operations leadership to get real-time issues addressed and resolved. There was a lot of questions that we always had. I think that was all very helpful and helped the division be dynamic and kind of adapt to different issues as they came up, specifically with a lot of these patients being under insured or not insured at all, or undocumented.” (APP, Interview/FG 12) “Some days, it was just like, "Oh, I missed an email, everything will change.” (Nurse Manager, Interview/FG 22)
**Importance of choice in COVID care – sense of autonomy/control over one’s work**	“I think the only other kind of weird thing about this COVID time was the fact that there were COVID providers and non-COVID providers, and it created this kind of weird separation in our group or like I don’t want to judge someone that’s not comfortable doing COVID, and I like the fact that our division respects people’s health and their ability to take care of COVID patients or not, but I think it also created – it creates this kind of weird dynamic… I think everybody respected people’s privacy, and – but you know who’s not a COVID provider.” (APP, Interview/FG 3)

*APP* Advanced Practice Provider

*FG* Focus Group

*MD* Doctor of Medicine

*DO* Doctor of Osteopathic Medicine

Dynamic clinical experience with a lot of uncertainty (Table 1). Clinicians, social workers, care management staff, and nursing staff and managers described situations of extreme uncertainty that ranged from clinical care to staffing models. Several subthemes are highlighted below.

### Surge staffing and ensuring sufficient workforce

Team members noted that patients with COVID-19 increasingly became more complicated as the pandemic continued. There were language barriers and barriers from personal protective equipment (PPE) limited the ability to communicate effectively. Patients with COVID-19 were often noted to be non-English speaking and with patients and families separated, there was a lot of additional work to help with communication. Participants noted that structures needed to be in place to help alleviate these additional duties or to have these roles be fulfilled in part by other care team members allowing physicians and APPs to focus attention on patient care.

APPs were felt to be integral to the COVID-19 response and were often considered experts in the care of patients with COVID-19 given the considerable amount of time they spent on the service. As the COVID-19 census grew and teams increased their ability to care for more and more patients, APP independent visits (as appropriate) were deployed (i.e. visits that were primarily conducted by the APP with physician back up support as needed). Although this adaptation was noted to be a cultural shift for the group, APPs felt that there was increased independence and physicians felt that the care model was safe and helped to ensure manageable workloads. There was some attending practice variation in this model (i.e. how much the attending would participate in the care of the APP visits) which required open communication and collaboration between various providers.

As patient numbers increased, staffing became more challenging. Moonlighting was utilized as a strategy to help increase staffing, but was felt to be a double edged sword; very helpful with last minute staffing needs but also contributed to a sense of obligation to work extra when faculty were already physically tired and emotionally exhausted. Ensuring that faculty did not feel they were required to work additional shifts was important but needed to be explicitly stated.

The COVID-19 surge brought rapid adaptations to teams. Specific teams were created to care for patients with COVID-19 and each week scheduling changes would occur in order to operationalize these specific teams (and to ensure providers were able to care for these patients). In an effort to minimize stress and constant changes, any changes in the schedule were prioritized to those who were already on service (i.e. not changing the specific day someone was assigned to work but instead possibly changing their assignment for the day). Minimizing changes, ensuring additional providers were not brought on unexpectedly, and building robust surge plans that explicitly stated next steps helped to build the necessary mental frameworks for what was going to happen next.

### Role delineation and ensuring appropriate training/working to top of license

As hospitalists shifted to the intensive care unit, initially there was a lack of role delineation; however, as the first surge progressed, the hospitalists eventually staffed their own team with intensivist consult support. This role delineation helped hospitalist clinicians to have appropriate autonomy, but also sufficient support from the intensive care unit specialists. Similar issues were faced around roles of consultants and clinical trials.

### Information sharing – centralized hubs and colleagues as resources

There was a flood of information and resources related to new processes and procedures including evolving best practices for the care of patients with COVID-19. Participants described access to this information as very helpful; however, not all of these resources were in one location. Human support was an important aspect of information sharing with reliance on those who had spent more time on services caring for patients with COVID-19 or those who were focusing on collecting and collating information. Team members on service relied on those individuals with the most experience, such as APPs.

### Importance of choice—autonomy/control over one’s work

From the very beginning of the pandemic, hospitalist clinicians were able to determine whether they felt they could care for patients with COVID-19 (i.e. providers may have had health conditions that precluded the exposure to COVID-19). Providers expressed gratitude for the option of choice, although they also expressed that the contributions of “non-COVID-19” providers could have been better recognized given the emphasis tended to be on those providing care for patients with COVID-19. While overall this approach of allowing providers to choose was felt to be helpful, it was noted that it could create challenging dynamics such that non-COVID providers may feel less recognized or can create hard feelings when a provider is a COVID provider and taking on more risk than others.

The importance of visible leadership with a focus on sense-making and clear communication strategies (Table 2).

**Table 2:** Importance of visible leadership with a focus on sense-making subthemes and exemplar quotes

**A unique dichotomy between flexibility and structure needed in a dynamic environment**	“It was very helpful as a team of COVID providers because we would all just still show up, sit at the conference room and sort of said, “How are your patients doing? Like what did you do? How – what have you been doing?” We all sort of – because day to day everything was changing so much, it was nice to just do like a little check-in and that’s how I feel like that sort of progressed, that it was more of a check-in with your teammates. You were working with an attending but then you still had three or four other COVID teams and we all got to sit around and just be like how are your patients doing? Oh, you sent two to MICU? We’re sorry.” (APP, Interview/FG 5) “There was a small pool left that kind of ended up working a lot the first few weeks, and so I feel like we kind of all had this shared experience with trying to figure out how to take care of these patients and trying to talk with our patients and see if there were similarities or trends that we were seeing, and so I think clinically, it was like this kind of scary but bonding experience.” (APP Interview/FG 6) “The nice thing is if there is any sort of issue or any sort of concern to just bring it to the-the appropriate level. So for instance, we would sometimes hear about something that had gone straight up, like through the command center to the C suite, and then also – and then it gets put back down to us. And had we just been able to have a direct conversation about it, I think it really could have easily been solved, because ultimately, it ends up coming back down to us to—to work on anyway. We just noticed that a few times where we're like, “well, we didn't even know this was a problem but, you know, we're happy to work on it.”” (Care Management, Interview/FG 9)
**Communication was key to effective operational response (transparent, consistent, concise)**	“There was a lot of communicating. It was good to hear from everyone and I liked the updates. It helped keep my anxiety at bay but at the same time, it may be made my anxiety worse sometimes because it was so much communicating.” (APP, Interview/FG 2) “I think that tiering system [tiered surge plan] is great because one of the most unsettling things, is uncertainty, and not knowing when we may scale up, scale down, can be unsettling. So, knowing, “Okay, so these are the criteria that moves us to tier 2, these are criteria that moves as to tier 3…” (MD/DO, Interview/FG 25)
**Sense of mission and increased collaborations**	“I think one of the biggest positives was a sense of an overarching mission within the group and seeing my colleagues step up … And so it was, it felt like a pretty important shift and right from our first meetings, kind of those first emergency meetings, there was a real sense of solidarity and mission within the group that really meant a lot to me as the first year within the group. And so that was cool to see. And it made me feel good about coming to work and good about signing up for extra shifts and good about everything that might happen, because I trusted the people that I was working with. (MD/DO, Interview/FG 1) “The sense of one team extending like well beyond anything I've seen before. The fact that we agreed to take obstetric COVID patients, that surgeons were interested in participating in the care planning, the fact that we were working with infectious diseases, that we had outpatient doctors who wanted to come and [help]. I mean, it was, it was, it was beautiful. That was a beautiful thing. So, I think it was probably the best crisis we could have, because we've established new relationships and approaches that never would have existed were it not for COVID.” (MD/DO, Interview/FG 20) “I found working on the COVID services to be incredibly rewarding and one of the most rewarding clinical experiences that I've had in a really long time. And part of that was the people that you were working with, whether it was with nursing staff, respiratory therapists, specialists that you were consulting; everyone was sort of in this like, "Hey, this is a catastrophe that's happening, it's a disaster, no one knows what the right answers are, and we're just trying to figure it out day to day. So it's taking creativity and working together as a real team. I think it was the most robust team feeling that I've ever had in a hospital.” (MD/DO, Interview/FG 18)

*MICU* medical intensive care unit

*APP* Advanced Practice Provider

*FG* Focus Group

*MD* Doctor of Medicine

*DO* Doctor of Osteopathic Medicine

#### A unique dichotomy existed between the flexibility and structure needed in a dynamic environment

Because the clinical environment was relatively unstable, creating structures around leadership, communication, and surge plans was needed, but there was also importance placed on embracing flexibility. Structure was needed in order to have a clear and unified plan, yet there were also natural forms of management/practices that organically emerged such as how APPs and physicians were working together and communication collaborations that emerged from talking to each other as sources of knowledge. Participants described the importance of both structure and flexibility.

There were a variety of leadership structures, including those of the hospitalist team, those of the department and school (i.e. next levels of leadership structure), and the command center structure of the hospital [5, 13]. Closed loop communication strategies (such as summary communications from leadership regarding the issues that had been addressed each day) were also developed that eliminated some of the unnecessary redundancy of communications and also highlighted items that had been successfully addressed. It was also noted that it was helpful to have clinicians in high-level leadership roles interface with the care teams (i.e. point person in the command center being a clinician). Having an available leadership team and robust feedback loops in place helped providers feel heard and engaged in the efforts to improve delivery of care during the pandemic.

#### Communication was key to effective operational response (transparent, consistent, and concise)

Communication from leadership was felt to be important, but it was noted that reasons behind decision-making (i.e. sense-making) should be made apparent.

Being mindful of the quantity of messaging was also felt to be important. Initially there was a flurry of emails coming from many different entities making it challenging to keep up. This communication strategy was quickly modified to a once-daily email with all communications being routed through and summarized by the hospital medicine clinical operations team. As the surge lessened, the frequency of these emails also decreased. Team touch points were created such that one individual from the clinical operations team would attend, functioning as the conduit between the teams and the command center. Having a point of contact with the clinical operations team was felt to be critical.

#### Shared sense of mission with increased collaborations

Participants described a sense of a common mission that was a powerful and uniting force. Team members felt that the collaborations and shared mission also helped to overcome many institutional barriers, resulting in problems being solved in real-time. Many felt that they had to fight the system less than usual. Care management and social work began to attend team huddles, which helped with information dissemination as well as correcting misinformation and misunderstandings.

There was significant emotional toll on individuals and their families (Table 3)

**Table 3:** Emotional toll subthemes and exemplar quotes

**Impact on the individual**	“I think that there was a lot of especially because COVID is hitting kind of racial and socio-economic groups and ethnic groups very differently. So, I felt like when I talked to my colleagues, they were sensing that a lot, like, you know, taking care of Nepalese families, and like, the whole family is hospitalized and like the two kids are at home. So, they're trying to figure out obviously, how to care for these patients, but also dealing with that burden and the stress and the moral distress you have of like seeing these health inequities in your face.” (MD/DO, Interview/FG 24) “I think clinically, like it was just such an unknown. There’s so much like fear stuff in the media and so you’re trying to stay informed, but I would – I couldn’t listen to the news driving into work because it would just get me stressed out like before I got here. So I think it was kind of interesting, thinking like wanting to stay in touch but then not wanting to let it get into you mentally before you come here and try and care for patients.” (APP, Interview/FG 6) “Nobody, nobody I've talked to in the city or in other states did have face respirators for their providers. Like, literally nobody. And from my perspective, I appreciated that, especially early on, because I had, I felt like I knew that no matter what happened, I had the equipment that I needed.” (MD/DO, Interview/FG 1) “This was so hard for everybody, I mean, I walk on those units, and the battle fatigue with the doctors and nurses and then the social workers and-and care managers as well. Um, I don't know what the answer is to that to kind of help us do better with the battle fatigue, but that all of those things, I think, just really took a toll.” (Care Management, Interview/FG 8) “I mean I really have to push through it. At the beginning I was super afraid. I even told my husband, "I don't know if I'm going to get out of this. I may get infected and I will die within the field.” (MD/DO, Interview/FG 21)
**Impact on personal life/family**	“I think that overarching stress, and then repeatedly having to go into an environment where you were, you know, being exposed, and then potentially exposing your family. And then experiencing, the medical care system in this new, dynamic environment. And not really being able to fully express that to a spouse who—who isn't experiencing it. I think was a little bit challenging and I think—I mean I think that universally would be true. It's kind of like going to med school. You can talk about it, but you can't fully explain—you can tell somebody who has been through it, they don't really know—know what that is like.” (MD/DO, Interview/FG 26) “It was tricky, because I mean personally, our family was newly pregnant during that time. And so I was like, “Oh, should I go to the ICU? Because there's a pregnant patient in ICU.” It's like, not looking so hot right now, and is this what happens to pregnant people? So there was just a lot of worry there…” (Interview)^a^

*APP* Advanced Practice Provider

*FG* Focus Group

*MD* Doctor of Medicine

*DO* Doctor of Osteopathic Medicine

^a^Workforce group, focus group not specified to maintain confidentiality

#### Impact on the individual while in the workplace

Participants commented that the experience was traumatic, taking an emotional toll on the individual, and that it is important to recognize this. It is also important to highlight the different support mechanisms in place. Ensuring there are different outlets for support helped team members to find support in different ways (i.e. virtual happy hours, focus groups, services offered by psychiatry). Interviewees noted a wide range of emotions including guilt when not on service, trauma, exhaustion, and even exhilaration.

Stress was alleviated and wellness was improved when there was more certainty provided with scheduling, there were resources to help manage home life/child care, and having colleagues to serve as outlets to talk through the emotional turmoil. Small tokens of gratitude were appreciated such as having food on the wards and people to check-in with you. Connection to mental health resources was also appreciated. Interviewees reported feeling grateful to have personal respirators and felt that safety was prioritized, which helped with anxiety and fear as the personal protective equipment early in the response was felt to be a source of stress given the many unknowns. Interviewees noted that it is helpful to set the culture/expectation that it is ok if other academic and administrative responsibilities are put on hold.

#### Impact on personal life/family

Providers described for the first time being fearful of going to work and an intense worry that they or their families would be at increased risk. Some families were expecting parents and that added additional stressors.

### Best practices

Table 4 highlights the best practices that came from the various interviews.

**Table 4:** Best practices

Theme	Best practice
**Dynamic clinical experience with uncertainty**	
Surge staffing and sufficient workforce	Clear proactive surge plans – with next steps for scaling up/down; shared broadly Predictable plans (as much as possible) with naming conventions of services that make sense Clear role delineation Geographically based teams COVID specific teams Translator phones/tablets in every room Consider language specific teams (i.e. care team fluent in predominant language) Ensure sufficient personal protective equipment Consider shorter shift stretches
Role delineation and ensuring appropriate training	Robust onboarding – ensuring that care teams have two main skill sets (operational knowledge, clinical skillset) Single point person for clinical trials to coordinate and help with unbiased approach to trials
Information sharing – centralized hubs and colleagues as resources	Allow for organically formed information hubs Ensure information in stored in single site, ideally synthesized and up to date
Importance of choice	Allow for choice in COVID care and recognize life situations may change choice Recognize all team members (those providing care for patients with COVID and those not) Follow closely the impact of types of services to try to ensure opportunities such as teaching are shared
**Importance of visible leadership with a focus on sensemaking**	
Unique dichotomy between flexibility and structure in environment	Embrace both grassroots efforts and more structured efforts Ensure more structured efforts do not inhibit communication or lead to mistrust
Communication is key to effective operational response	Rounding on the floors regularly Ensure teams know it is ok to take a break Team lead to communicate frontline concerns and provide loop closure Help teams to understand the reasoning behind decisions Ensure sense-making when discussing financials Minimize other pressures (i.e. academic responsibilities) Standardized forms of communication Limit the quantity of communications (i.e. batch) If multiple institutions ensure congruent messaging Help with sense-making Variety of formats (zoom, email, team touchpoints) Ensure clinical liaisons for command center and single point person for frontline teams Go to the area of concern instead of going several leadership levels above When in active new situation, afternoon in person huddles where appropriate (small groups)
Sense of mission and increased collaborations	Small tokens of gratitude such as food, notes, parking, communications expressing gratitude Build wellness into surge plans – ensuring plans are visible Address child care Small ways to connect – zoom happy hours, outreach platforms Lower administrative barriers (documentation, mask centers)
**Significant emotional toll**	
Impact on individual in the workplace	Ensure support systems such as focus groups and mental health services Send clear messaging that when you are off it is ok to be off Ensure sufficient staffing Ensure team understands that it is ok that academic endeavors may lag
Impact on personal life/family	Ensure sufficient and safe PPE Foster access to infectious disease specialists to answer questions and provide support Prioritize predictability in scheduling as much as possible (type of work may change, but shifts, hours, duration of the day will be predictable) Offer employee benefits for caregiving duties

*PPE* personal protective equipment

## Discussion

Three themes emerged from the work including (1) dynamic clinical experience with a lot of uncertainty (2) the importance of visible leadership with a focus on sense-making and (3) the significant emotional toll on care team members. The pandemic and the resulting adaptations impacted the workforce both professionally and personally. Several best practices emerged from the work that were felt to help mitigate some of the uncertainty and emotional toll placed on clinical care teams.

A variety of adaptations during COVID-19 have been described in the literature [6, 14] and include varying approaches (i.e. lower census versus higher census, geographically based respiratory isolation units versus no geography and many modifications thereof). This study represents one of the first studies to assess the impact of adaptations on frontline care teams. By utilizing rapid qualitative techniques, we were able to assess in real-time surge efforts. Following the results of this study, we were able to iteratively modify the adaptations for future surges by keeping the helpful adaptations and modifying those that seemed to be less successful. Rapid qualitative methods represent one methodologically sound way to quickly assess operational activities in hospital medicine with minimal cost and a much more rapid timeline than typical qualitative work.

COVID-19 has had both physical and emotional effects on the frontline workforce [15–19] and this qualitative work further highlighted these issues. Previous work characterizing anonymous physician perspectives about the experience of COVID-19 highlighted issues around trust and concerns around PPE [20]. Early in the pandemic, the PPE situation was of concern across the country, however, we were able to quickly remedy the situation with half face mask respirators [21, 22] for the entire team caring for patients with COVID-19.

Previous work has highlighted that burnout is common in the field of hospital medicine [23]. There is a need to further our understanding of what drives the baseline burnout as well as how these additional tolls can be mitigated as many of the issues raised during the pandemic highlight on a larger scale the baseline issues that existed well before the pandemic. Ensuring safe and sustainable staffing models that benefit patients, providers, and institutional goals should be sought as these are foundational to all of our efforts [24, 25]. At baseline, our healthcare systems do not typically prioritize attention in the workplace and the pandemic and its surges have brought about even more chaos in our clinical environments. Adding insult to injury, hospital systems are now facing staffing shortages. Hospital medicine will continue to grapple with these issues as payment models evolve [26] and hospitals face financial uncertainties [27]. Higher patient loads and capacity related issues have been shown to negatively impact quality improvement efforts [28], throughput [29, 30], and ultimately patient care [31–35] and thus understanding optimal staffing strategies is imperative.

An additional source of stress has been trying to find an equivalency for the skill set that a hospitalist team brings – that of an operational expert paired with inpatient medical expertise. Our team on-boarded outpatient providers and paired them with residents on inpatient wards, moving hospitalists from teaching teams to direct care of patients with COVID-19 in partnership with an APP. During the second surge, processes were built to take advantage of naturally occurring teams and were felt to be better options than calling in outpatient providers who may have to cancel clinics. As an example, a medicine sub-specialty team could take patients with COVID or broaden diagnoses that the team may typically take, utilizing existing teams and team members already in the hospital during the day and potentially at night.

### Strengths

Our study has several strengths. This work represents one of the first scientific works highlighting the experience of clinicians, care managers, social workers, and nurse managers with the first COVID-19 surge as well as an analysis of the various tactics utilized. We utilized rapid qualitative methods to allow for real-time analysis of this work to inform our current surge plans. Fields that need rapid acquisition of data may be particularly interested in rapid qualitative methods to generate preliminary data for funding opportunities, understand complex and dynamic operational processes, to evaluate new programs (clinical, educational, or otherwise), or in process improvement work, adding to the traditional quantitative data that are more commonly utilized.

### Limitations

Our study also has several limitations, including being a single center study. There were several topics not raised during our interviews that have been previously noted to be issues on social media platforms such as the lack of salary increases, salary freezes, and furloughs, as well as hiring freezes. We believe that these are issues that may evolve to be of greater importance as the pandemic continues; however, our focus groups and interviews focused on operational issues and thus that may have also been why these issues were not raised by participants. Some meanings of the transcripts from the interviews might have been lost. To mitigate this, we did conduct member checking with participants to ensure the results were credible and reflected the lived experience of the participants. No additional revisions were suggested by the participants that reviewed the findings. In addition to the transcripts, field notes were taken to add additional context. We utilized rapid qualitative methods, which may not result in discovery of more subtle findings. This work did not include quantitative outcomes which will be important for future work, pairing both quantitative and qualitative methods for a more robust understanding of surge practices and their impact. Finally, this work was considered program evaluation and thus not generalizable to other settings.

## Conclusion

COVID-19 surge practices have direct impact on hospitalist providers and support teams. Transparency, frequent but strategic communications, and sufficient workforce support are key factors for successful surge planning. Rapid qualitative methods allowed for speedy acquisition of data to understand how adaptations were experienced by frontline care teams during a rapidly evolving clinical situation. The findings learned from this work have already resulted in new implementations (or de-implemented strategies). As institutions rapidly evolve their surge strategies for the ongoing pandemic, ensuring the impact of the adaptations are assessed will be key to optimizing the various initiatives deployed.

## Supplementary Information

**Additional file 1.**

**Additional file 2.**

## Data Availability

The datasets generated and/or analyzed during the current study are not publicly available due to the fact that this work is qualitative in nature and while doesn’t include identifiers it does include information that within certain contexts could be perceived as identifiable. The data are available from the corresponding author on reasonable request and upon approval from our research board/regulatory bodies.

## Abbreviations

APPs: Advanced practice providers (physician assistants and nurse practitioners)
COVID-19: Coronavirus disease 2019
FG: Focus Group
MD: Doctor of Medicine
MICU: Medical intensive care unit
DO: Doctor of Osteopathic Medicine
PPE: Personal protective equipment

## References

[1] Bowden K, Burnham EL, Keniston A, et al. Harnessing the power of hospitalists in operational disaster planning: COVID-19. J Gen Intern Med. 2020;35:2732–7. 10.1007/s11606-020-05952-6 PMC7358298 32661930

[2] Garg M, Wray CM (2020). Hospital Medicine Management in the Time of COVID-19: Preparing for a Sprint and a Marathon. J Hosp Med.

[3] Kim MK et al. A Primer for Clinican Deployment to the Medicine Floors from an Epicenter of COVID-19. NEJM Catalyst. May 4, 2020. https://catalyst.nejm.org/doi/full/10.1056/CAT.20.0180 Accessed February 12, 2021.

[4] Biala D, Siegel EJ, Silver L, Schindel B, Smith KM. Deployed: Pediatric Residents Caring for Adults During COVID-19’s First Wave in New York City. J Hosp Med. 2020;15:763–4. 10.12788/jhm.3527 33231546

[5] Persoff J, Ornoff D, Little C (2018). The Role of Hospital Medicine in Emergency Preparedness: A Framework for Hospitalist Leadership in Disaster Preparedness, Response, and Recovery. J Hosp Med.

[6] Auerbach A, O’Leary KJ, Greysen SR, et al. Hospital Ward Adaptation During the COVID-19 Pandemic: A National Survey of Academic Medical Centers. J Hosp Med. 2020;15:483–8. 10.12788/jhm.3476 PMC7518133 32804610

[7] Hamilton A. Qualitative methods in rapid turn-around health services research. Health Services. Research & Development Cyberseminar. 2013 Dec 11. Available at: https://www.hsrd.research.va.gov/for_researchers/cyber_seminars/archives/780-notes.pdf. Accessed February 12, 2021.

[8] Averill JB (2002). Matrix analysis as a complementary analytic strategy in qualitative inquiry. Qual Health Res.

[9] Vindrola-Padros C, Chisnall G, Cooper S (2020). Carrying Out Rapid Qualitative Research During a Pandemic: Emerging Lessons From COVID-19. Qual Health Res.

[10] Moreau J. Rapid Qualitative Methods. Colorado Pragmatic Research in Health Conference. August 12, 2020. Aurora, Colorado.

[11] Elo S, Kyngäs H (2008). The qualitative content analysis process. J Adv Nurs.

[12] Birt L, Scott S, Cavers D, Campbell C, Walter F (2016). Member Checking: A Tool to Enhance Trustworthiness or Merely a Nod to Validation?. Qual Health Res.

[13] Tevis SE, Patel H, Singh S, et al. Impact of a Physician Clinical Support Supervisor in Supporting Patients and Families, Staff, and the Health-Care System During the COVID-19 Pandemic. Disaster Med Public Health Prep 2020:1–5. 10.1017/dmp.2020.345 PMC7653485 32907663

[14] Linker AS, Kulkarni SA, Astik GJ, et al. Bracing for the wave: a multi-institutional survey analysis of inpatient workforce adaptations in the first phase of COVID-19. J Gen Intern Med. 2021;36:3456–61. 10.1007/s11606-021-06697-6 PMC8161717 34047919

[15] Jones Y, Durand V, Morton K (2020). Collateral Damage: How COVID-19 Is Adversely Impacting Women Physicians. J Hosp Med.

[16] Daubman BR, Black L, Goodman A (2020). Recognizing Moral Distress in the COVID-19 Pandemic: Lessons From Global Disaster Response. J Hosp Med.

[17] Morganstein JC. Web Exclusive. Annals for Hospitalists Inpatient Notes - Preparing for Battle: How Hospitalists Can Manage the Stress of COVID-19. Ann Intern Med 2020;172:HO2-HO3. 10.7326/M20-1897 32315380

[18] Norful AA, Rosenfeld A, Schroeder K, Travers JL, Aliyu S (2021). Primary drivers and psychological manifestations of stress in frontline healthcare workforce during the initial COVID-19 outbreak in the United States. Gen Hosp Psychiatry.

[19] Dugani SB, Geyer HL, Maniaci MJ, Fischer KM, Croghan IT, Burton C (1995). Psychological wellness of internal medicine hospitalists during the COVID-19 pandemic. Hosp Pract.

[20] Sullivan K, Burden M, Keniston A, Banda J, Hunter LE (2020). Characterization of Anonymous Physician Perspectives on COVID-19 Using Social Media Data. Pac Symp Biocomput Biocomputing.

[21] Bessesen MT, Adams JC, Radonovich L, Anderson J (2015). Disinfection of reusable elastomeric respirators by health care workers: a feasibility study and development of standard operating procedures. Am J Infect Control.

[22] Pompeii LA, Kraft CS, Brownsword EA (2020). Training and Fit Testing of Health Care Personnel for Reusable Elastomeric Half-Mask Respirators Compared With Disposable N95 Respirators. JAMA.

[23] Roberts DL, Shanafelt TD, Dyrbye LN, West CP (2014). A national comparison of burnout and work-life balance among internal medicine hospitalists and outpatient general internists. J Hosp Med.

[24] Arogyaswamy S, Vukovic N, Keniston A, et al. The Impact of Hospital Capacity Strain: a Qualitative Analysis of Experience and Solutions at 13 Academic Medical Centers. J Gen Intern Med 2021. 10.1007/s11606-021-07106-8 PMC8667526 34902096

[25] Kissler MJ, Kissler K, Burden M. Toward a Medical “Ecology of Attention.” N Engl J Med. 2021;384:299–301. 10.1056/NEJMp2027190 PMC8114173 33503689

[26] Society of Hospital Medicine. Policy Update: Cuts to Hospitalist Medicare Reimbursement in 2021. https://www.hospitalmedicine.org/policy--advocacy/letters/pfs-2021-Medicare-Reimbursement-Cuts/ Accessed February 13, 2021.

[27] American Hospital Association. Hospitals and Health Systems Face Unprecedented Financial Pressures Due to COVID-19. https://www.aha.org/guidesreports/2020-05-05-hospitals-and-health-systems-face-unprecedented-financial-pressures-due. Accessed February 13, 2021.

[28] Kara A, Flanagan ME, Gruber R (2020). A Time Motion Study Evaluating the Impact of Geographic Cohorting of Hospitalists. J Hosp Med.

[29] Elliott DJ, Young RS, Brice J, Aguiar R, Kolm P (2014). Effect of hospitalist workload on the quality and efficiency of care. JAMA Intern Med.

[30] Zoucha J, Hull M, Keniston A (2018). Barriers to Early Hospital Discharge: A Cross-Sectional Study at Five Academic Hospitals. J Hosp Med.

[31] Kulstad EB, Sikka R, Sweis RT, Kelley KM, Rzechula KH (2010). ED overcrowding is associated with an increased frequency of medication errors. Am J Emerg Med.

[32] Richardson DB (2006). Increase in patient mortality at 10 days associated with emergency department overcrowding. Med J Aust.

[33] Hoot NR, Aronsky D (2008). Systematic review of emergency department crowding: causes, effects, and solutions. Ann Emerg Med.

[34] Singer AJ, Thode HC, Viccellio P, Pines JM (2011). The association between length of emergency department boarding and mortality. Acad Emerg Med.

[35] Pines JM, Iyer S, Disbot M, Hollander JE, Shofer FS, Datner EM (2008). The effect of emergency department crowding on patient satisfaction for admitted patients. Acad Emerg Med.

