# Supplementary figures and images for: The impact of surge adaptations on hospitalist care teams during the COVID-19 pandemic utilizing a rapid qualitative analysis approach
Source: Arch Public Health. 2022 Feb 17;80:57. doi: 10.1186/s13690-022-00804-7 (PMC8851813; doi:10.1186/s13690-022-00804-7)

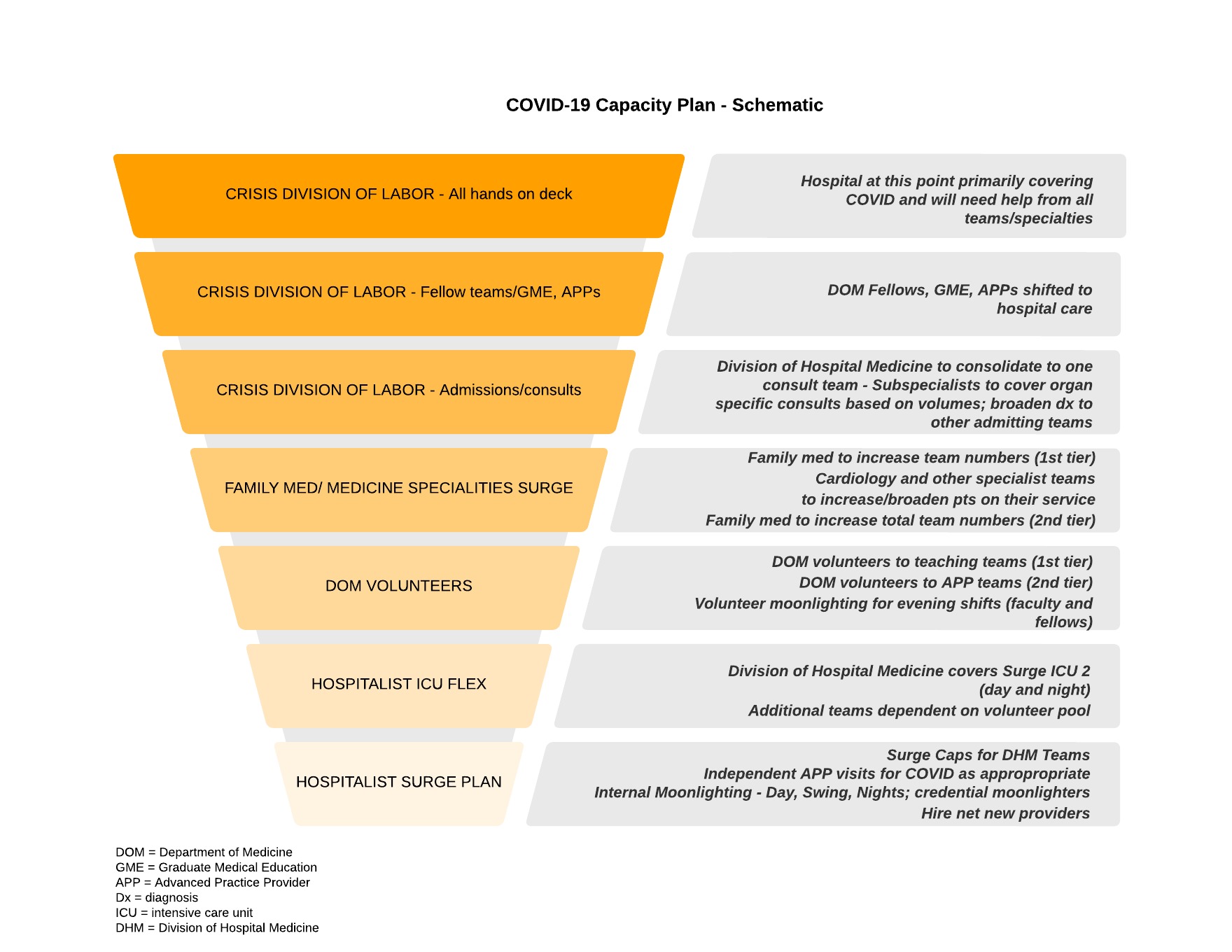

Supplement: Supplementary file 1 — Additional file 1. [file 13690_2022_804_MOESM1_ESM.jpeg]
