# Supplementary material for: The impact of surge adaptations on hospitalist care teams during the COVID-19 pandemic utilizing a rapid qualitative analysis approach
Source: Arch Public Health. 2022 Feb 17;80:57. doi: 10.1186/s13690-022-00804-7 (PMC8851813; doi:10.1186/s13690-022-00804-7)
Supplement: Supplementary file 2 — Additional file 2. [file 13690_2022_804_MOESM2_ESM.docx]

**Appendix 2.**

**Before we get started I wanted to discuss some ground rules for our conversation today.**

First there are no right or wrong answers, only differing points of view. You don't need to agree with others, but we ask that you listen respectfully as others share their views. Please feel free to share your point of view even if it differs from what others have said. Keep in mind that we're just as interested in negative comments as positive comments.

My role as moderator will be to guide the discussion and make sure that everyone has a chance to speak but this is a conversion between all of us in the group so please feel free to talk to each other. As I mentioned previously, we will be recording this session. If you are uncomfortable being recorded, please let me know.

Please turn off your cellphones and make sure you are in a place where you will not be interrupted. Does anyone have any questions before we begin?

**Guided Questions Script- Hospital Medicine Faculty on COVID-19 Teams**

**#1 To get started, I’d like to know a little more about you. What has your role been during COVID-19?**

Probe: What types of services have you worked on? (COVID teams, non-COVID teams, ICU teams, etc.)

Probe: How did you prepare for those roles?

Probe: How did you feel working in these roles?

Probe: What types of support did you have when in these roles (examples might be clinical care guidelines, support on best practices for PPE use, for those in ICU roles support of folks with that skill set)?

**#2 Can you walk me through your experience during the pandemic, specifically focusing on your clinical work and how our team surged (or how you experienced the surge)?**

Probe: What worked well? What didn’t work well? Why?

Probe: How have the surge practices affected you?

Probe: How do you think have the surge practices affected our patients?

Probe: What else did you notice during all of these changes?

**#3 During this very dynamic time, what did you notice about communication?**

Probe: How did the surge affect collaboration and communication between different teams and departments?

Probe: How present were leadership teams and members in facilitating coordination, collaboration, and communication?

Prompt: Can you describe communications and collaborations with our hospital colleagues?

Prompt: What did you notice about communication and collaboration for clinical trials?

Probe: What recommendations do you have to better facilitate communication, coordination, and collaboration?

**#4 Can you tell me how COVID-19 surge practices affected you personally?**

Probe: How was your personal wellness affected?

Probe**:** How can COVID-19 surge practices better address/incorporate wellness?

**#5 What recommendations do you have to ensure optimal COVID-19 surge capacity management going forward?**

Probe: Can you tell me more about these recommendations and how they could be implemented?

Probe: What challenges do you think the hospital or our group may face implementing these best practice recommendations?

**Anything I didn’t ask you about that you think is important for me to know?**

**Is there anyone you would recommend I also interview?**

**----**

**Guided script for care management, social work, nursing, and hospital leadership**

**#1 To get started, I’d like to know a little more about you. What has your role been during COVID-19?**

Probe: In what ways did you interact with the hospitalist team during the pandemic?

**#2 During the first wave of the pandemic, there were numerous structural changes made by the front line teams including hospitalists, care management, social work, and nursing. Can you tell me about your experience with those changes?**

Probe: Can you tell me what your interactions and experience with hospitalist leadership and teams was like?

Probe: What worked well and what could be better?

**#3 As we aim to improve our collaborations with our key partners such as yourself, what would you like to see us continue to do? And what would you like to see changed?**

Probe: How was our communication?

Probe: What was your perception of our team structures?

Probe: How would you describe the collaboration?

**#4 Anything I didn’t ask you about that you think is important for me to know?**

**# 5 Is there anyone you would recommend I also interview?**
